# Retraction: Harnessing the power of AI: Advanced deep learning models optimization for accurate SARS-CoV-2 forecasting

**DOI:** 10.1371/journal.pone.0321233

**Published:** 2025-04-09

**Authors:** The PLOS One Editors

The *PLOS One* Editors retract this article [1,2] due to concerns about peer review integrity, authorship, and adherence to the journal’s publication requirements on reporting and data availability. Among other concerns, most of Table 1 and Figs 2–10 were subsequently published in [3,4], raising concerns about redundant publication. We regret that the issues were not addressed prior to the article’s publication.

MUT, SBI, and MB did not agree with the retraction. AA either did not respond directly or could not be reached.
